# Variability in the protein profiles in spermatozoa of two sturgeon species

**DOI:** 10.1371/journal.pone.0186003

**Published:** 2017-10-27

**Authors:** Ping Li, Wei Guo, Huamei Yue, Chuangju Li, Hao Du, Xinmei Qiao, Zhigang Liu, Qiong Zhou, Qiwei Wei

**Affiliations:** 1 Key Laboratory of Freshwater Biodiversity Conservation, Ministry of Agriculture of China, Yangtze River Fisheries Research Institute, Chinese Academy of Fishery Sciences, Wuhan, China; 2 Sino-Czech Joint Laboratory for Fish Conservation and Biotechnology, Yangtze River Fisheries Research Institute, Chinese Academy of Fishery Sciences, Wuhan, China; 3 University of South Bohemia in České Budějovice, Faculty of Fisheries and Protection of Waters, South Bohemian Research Center of Aquaculture and Biodiversity of Hydrocenoses, Research Institute of Fish Culture and Hydrobiology, Zátiší, Vodňany, Czech Republic; Universite Clermont Auvergne, FRANCE

## Abstract

Conventional sperm analysis (i.e., motility and fertility) has been used to evaluate sperm quality. Understanding the quality of sperm on the molecular level in the sturgeons, *Acipenser baerii* and *A*. *schrenckii*, is essential for the improvement of the conservation of genetic resources and farming performance. In this study, we used the iTRAQ proteomics approach to perform proteomic profiling of spermatozoa associated with sperm quality in sturgeons (Data are available via ProteomeXchange with identifier PXD006108). The results showed 291 and 359 differentially expressed proteins in *A*. *baerii* and *A*. *schrenckii*, respectively, of which 72 were common to both species and all were upregulated in high quality compared with low quality samples. The differentially expressed proteins were mainly categorized into the generation of precursor metabolites and energy and oxidation, and they were localized to the mitochondria. Three distinguishing pathways, Arginine and proline metabolism, Pyruvate metabolism and the Citrate cycle (TCA cycle) were found to play an important role in energy metabolism, and some substrates could be used in the sperm medium for storage and cryopreservation. The quantity levels of two proteins, CKMT1 and LDHB, were verified by western blot analysis. Moreover, other potential biomarkers involved in oxidation reduction, ubiquitin-proteasome-dependent proteolysis, chaperones and binding activity were also discussed. Our study is the first to use the iTRAQ-based proteomics approach to analyse the sturgeon spermatozoa proteome, and the results that we obtained are valuable for the prediction of sperm quality and reproduction management in these threatened species.

## Introduction

Sturgeons, as “living fossils”, belong to the order Acipenseriformes, which is one of the oldest in the class Actinopterygii [1]. The population of sturgeons was declined dramatically due to overharvesting, construction of dams and pollutions etc. and most of them are in endangered conditions. Moreover, the quality of sturgeon sperm is quite different among different males and even among different sperm from the same male. Studies of sperm biology could help improve reproduction and cryopreservation protocols of these endangered species.

Fish sperm quality could be reflected by some physiological indices, including a measurement of the seminal volume, the sperm concentration, the percentage of progressively motile and morphologically normal spermatozoa [2–3], besides of the fertilization rate. While the membrane integrity, mitochondrial function and chromatin structure integrity can also reflect the quality of sperm, especially widely used in the evaluation of effect of cryopreservation in sperm [4]. In recent years, the rapid technology development in detection of fish sperm quality, such as computer aided sperm analysis system (CASA), flow cytometry (FCM) and single cell gel electrophoresis (SCGE) makes the index more diversified and objective. But the evaluating method of fish sperm quality is still not perfect. Moreover, our previous studies showed that the sperm quality was quite different among the individuals after cryopreservation, even thought those samples were picked up as the "high quality sperm" according to traditional methods. The same phenomenon has been reported in mammalian sperm cryopreservation [5–6]. However, there is still information gap about the exact mechanisms of determining the quality and the fertilizing potential of sperm. Therefore, it is necessary to develop some new technologies or methods to evaluate the sperm quality.

As a structural basis for sperm cells to function, protein has become the focus of academic research, especially in the field of male reproductive medicine, and has achieved certain results [7–9]. Till now, some scientists have found several potential protein markers associated with male fertility in bulls [10–12] and stallions [13–14]. But the related studies in fish are lack. Furthermore, proteomic studies are more applied in the optimization of artificial fish reproduction and the development of cryopreservation techniques. The objective of this study was to investigate the global proteome of the spermatozoa of fertile *A*. *baerii* and *A*. *schrenckii* to determine if any of the proteins observed could be associated with a conventional sperm quality parameter. Common markers of higher and lower quality sperm in both species were also explored.

## Materials and methods

### Ethics statement

The experimental procedures were done following the criterions made by Hubei Province Association for Laboratory Animal Sciences, and approved by the Animal Care and Use Committee of the Yangtze River Fisheries Research Institute, Chinese Academy of Fishery Sciences.

### Collection and separation of sperm

Mature male and female *A*. *baerii* and *A*. *schrenckii* used in this study were collected from Hubei Hengsheng Industrial Co., Ltd. at Jingzhou and cultured in the Hatchery for Chinese Sturgeon, Yangtze River Fisheries Research Institute, Chinese Academy of Fisheries Science. Spermiation in males (11 and 7 individuals for *A*. *baerii* and *A*. *schrenckii*, respectively) was induced using an intramuscular injection of approximately 5 μg/kg of luteinizing hormone-releasing hormone A2 (LRH-A2) and 0.5 mg/kg domperidone (DOM). After 12 hrs, the sperm was obtained by gentle abdominal massage, taking special care to avoid blood, urine, or faecal contamination. Females were induced to ovulate first by an intramuscular injection of 5 μg/kg LRH-A2 24 h before stripping, and then by a second intramuscular injection of 9 μg/kg LRH-A2, 1 mg/kg DOM and 1 mg/kg of vitamin B1 12 h before stripping. The eggs were obtained by abdominal massage and stored in dry bowls. A sperm quality assessment was immediately performed, and it included motility and fertility.

Sperm motility parameters were measured immediately after sperm collection using a computer-assisted sperm analysis device (CASA, Leica DM2500, Germany; JVC TK-U890EG, Japan; FSQAS-2000, China) and sperm motility was activated with distilled water. The sperm motility parameters, including the A grade (move forward in a rapid and straight line, > 20 μm/s) percentage of motile sperm and curvilinear velocity (VCL), were measured over a four second period, between 10 and 14 s after activation. A mean of three repetitions was calculated per male.

A fertilization experiment was performed with the same batch of eggs. Each male fish in an experimental group fertilized 100 to 120 eggs, weighing approximately 0.4 g. The spermatozoa-to-egg ratio was 10^5^:1 (the sperm density was determined according to a haemacytometric method). All fertilization trials were performed in duplicate and the fertilization rate was measured at Stage 17 –the small yolk plug period, as the morphological characteristics of Stage 17 were easy to observe.

ANOVA was used to compare the mass motility and fertility between males. Differences were considered significant at p<0.05. High and low quality sperm were grouped according to the motility and fertility data.

### Protein extraction

The spermatozoa from each individual in a group were pooled together for protein extraction (samples of *A*. *baerii* and *A*. *schrenckii* were pooled from 4 and 3 individuals, respectively). The total protein was extracted using the cold acetone method. Ethylenediaminetetraaceticacid (EDTA—2 mM) and phenylmethanesulphonyl fluoride (PMSF—1 mM), dithiothreitol (DTT—10 mM) were added, and the samples were ground to disrupt the cells. The samples were centrifuged at 25,000 × g for 20 min at 4°C, and the pellets were discarded. DTT (10 mM) in 5 times the volume of cold acetone was added to the samples, followed by overnight incubation at −20°C and centrifugation at 25,000 × g at 4°C for 20 minutes, then the supernatant was discarded. To reduce the acidity, the pellet was washed with 10 mM DDT in 1.5 mL cold acetone and centrifuged at 25,000 × g at 4°C for 20 min. The acetone wash was repeated. The precipitate was dried in a vacuum concentrator for 5 min, and the dried pellet was lysed with 1 ml of protein extraction reagent [8 M urea, 4% (w/v) CHAPS, 30 mM HEPES, 1 mM PMSF, 2 mM EDTA, and 10 mM DTT] and sonicated for 5 min. The samples were centrifuged at 25,000 × g for 20 min at 4°C to remove non-soluble impurities. The protein concentration was determined with the 2-D Quant Kit (General Electric Company, USA). SDS-PAGE was performed to verify the protein quality and concentration.

### iTRAQ labelling

The iTRAQ labelling procedure was performed following the instructions provided in the iTRAQ labelling kit (Applied Biosystems), unless otherwise specified. For each protein sample, 100 μg of protein was denatured, and the cysteine residues were blocked as described in the iTRAQ protocol. The protein samples were digested with 5 μg of sequencing-grade modified trypsin (Promega, Madison, WI) at 37°C for 36 h. The digested samples were dried in a centrifugal vacuum concentrator, and the protein pellets were dissolved in 30 μL of 50% tetraethylammonium bicarbonate (TEAB) (Sigma, St. Louis, MO) together with 70 μL of isopropanol and labelled with the iTRAQ reagents according to the protocol of the 8-plex iTRAQ labelling kit. The trypsin-digested samples were analysed via matrix-assisted laser desorption/ionization time-of-flight/time-of-filight (MALDI-TOF-TOF) to ensure complete digestion. iTRAQ tags 113–121 were added to the digested protein samples during labelling. The iTRAQ-labelled samples were then pooled and subjected to strong cation exchange (SCX) fractionation.

### Strong cation exchange (SCX) fractionation

The labelled samples were fractionated using a high-performance liquid chromatography (HPLC) system (LC-20AB, Shimadzu, Japan) connected to an SCX column (Ultremex column, 4.6 mm I.D. × 250 mm, Phenomenex). The retained peptides were dissolved using 4 mL of buffer A (25 mM NaH_2_PO_4_ in 25% ACN, pH 2.7). After the peptides flowed onto the columns, the retained peptides were eluted using Buffer A for 10 min and 5–35% Buffer B (25 mM NaH_2_PO_4_, 1 M KCl in 25% ACN, pH 2.7) for 20 min, and then eluted using 35–80% buffer B for 1 min. The flow rate was set at 1 mL/min. Fractions were collected in 1.5 mL microfuge tubes every minute starting at 15 min after sample injection, and a total of 10 fractions was collected. The salt was removed from fractions with a high salt content using a Strata-X 33 μm Polymeric Reversed Phase column. The eluted fractions were dried in a vacuum concentrator and then dissolved in 0.1% formic acid prior to reverse-phase nLC-tandem mass spectrometry.

### Reverse-phase nanoliquid chromatography/tandem MS (LC-MS/MS)

The peptide content in each fraction was equalized prior to injection into the Nano-LC system. For the MALDI-TOF/TOF analysis, the SCX peptide fractions were pooled to obtain 17 fractions to reduce the peptide complexity. A 10 μL aliquot of each fraction was injected twice into the Proxeon Easy Nano-LC system. The peptides were separated using a C18 analytical reverse-phase column at a solvent flow rate of 300 nL/min (Solution A, 5% acetonitrile/0.1% formic acid; Solution B, 95% acetonitrile/0.1% formic acid) over 120 min. A linear LC gradient profile was used to elute the peptides from the column. After the sample was injected, the column was equilibrated with 5% Solution B for 10 min, and the following gradient schedule was initiated: 45% Solution B at 80 min; 80% Solution B at 85 min and maintained for 15 min; and 5% Solution B at 105 min and held for 15 min before ramping back down to the initial solvent conditions. The fractions were analysed using a hybrid quadrupole/time-of-flight MS (TOF-5600, Bruker, Germany) with a nanoelectrospray ion source. The data were collected and analysed using Data Analysis Software (Bruker Daltonics, Bremen, Germany). The MS/MS scans were recorded from 50–2000 m/z. Nitrogen was used as the collision gas. The ionisation tip voltage and interface temperature were set at 1250 V and 150°C, respectively.

### Data analysis

All of the mass spectrometry data were collected using Bruker Daltonics micrOTOF control and processed and analysed using the Data Analysis Software. The Uniprot database was downloaded and integrated into the Mascot search engine, version 2.3.01, through its database maintenance unit. The parameters were set as follows: trypsin was specified as the digestion enzyme, cysteine carbamidomethylation as a fixed modification, iTRAQ 8Plex on the N-terminal residue, iTRAQ 8Plex on tyrosine (Y), iTRAQ 8Plex on lysine (K), glutamine as pyroglutamic acid, and oxidation on methionine (M) as a variable modification. The tolerance settings for peptide identification in the Mascot searches were 0.05 Da for MS and 0.05 Da for MS/MS. The Mascot search results were exported into a DAT FILE and normalized and quantified using the Scaffold version 3.0 Software. Protein quantification was carried out based on a unique peptide. A cut-off of a 1.5-fold change was chosen, and proteins with quantification ratios of 1.5 for low (<0.67) or high (>1.5) relative protein levels were considered as differentially regulated.

### Functional analysis of differentially expressed proteins

To predict the functions of the differentially expressed proteins, we analysed the proteins in terms of three aspects. First, we categorized the proteins functionally using the WEGO (web Gene Ontology Annotation Plot) web service (http://wego.genomics.org.cn/cgi-bin/wego/index.pl). The consensus or exemplar sequences of the proteins were then subjected to BLAST searches against the appropriate database, and the top hits were selected using an E-value cut-off of 10^−5^. Next, we performed a functional category gene enrichment test using Blast2GO to determine whether the differentially expressed proteins were significantly enriched in any functional subcategories. An FDR significance threshold of 0.05 was selected. At last, we mapped the differentially expressed proteins to biological pathways using the Kyoto Encyclopedia of Genes and Genomes (KEGG) resource (www.genome.jp/kegg/).

### Western blot analysis

From the differentially expressed proteins, we selected Ckmt1 protein (CKMT1) and L-lactate dehydrogenase B-A chain (LDHB) involved in the Arginine and proline metabolism pathways, the TCA cycle and Pyruvate metabolism for western blot analysis to validate their quantity levels in high and low quality spermatozoa. Western blot samples were prepared in ice-cold RIPA containing 1 mM PMSF. The protein concentration was determined using the BCA assay kit (Beyotime, Shanghai, China). Equal amounts of total protein was separated by 12% SDS-PAGE and then transferred to an NC membrane (Milipore). After blocking with 5% non-fat milk at room temperature for 2 h, the membrane was incubated with primary antibody overnight at 4°C, (i.e., anti-LDHB 1:400; Atagenix, ATApla5470, rabbit; anti-CKMT1 1:400, Atagenix, ATApla8239, rabbit; or anti-β-actin 1:5000, Atagenix, ATA10167, mouse). After washing with TBST three times, the membrane was incubated with the corresponding secondary antibody conjugated to HRP (1:5000, Atagenix) for 1 h at room temperature. β-actin was used as an internal control. Western blot analysis was carried out using each sturgeon samples (three biological repeats), and Student’s *t*-test was used.

## Results

### Sperm motility and fertility parameters

The results for the sperm motility and fertilization rate are shown in Table 1. Clearly, the differences in the A grade percentage motility, VCL and fertilization rate of different males of *A*. *baerii* were high, ranging from 43.95±18.86% to 82.98±6.83%, from 71.56±4.76 to 142.73±11.83 μm/s and from 21.67±9.94 to 81.68±7.10%, respectively. For A grade motility, males 1, 2, 5, 6, 9 and 11 were placed in the low level group (43.95–57.18%), and the other males were placed in the high level group (males 3, 4, 7 and 10, 68.30–82.98%). For VCL, males 1, 2, 5, 6, 8, and 9 were placed in the low level group (71.56–85.55 μm/s), and the other males were placed in the high level group (males 3, 4, 7, 10 and 11, 96.76–142.73 μm/s). For the fertilization rate, males 2, 5, 6, 8, 9 and 11 were placed in the low level group (21.67–45.74%), and the others (males 1, 3, 4, 7 and 10) were placed in the high level group (65.99–81.68%). Combining all parameters under consideration, males 2, 6, 8 and 9 were placed in the low quality group, and males 3, 4, 7 and 10 were placed in the high quality group.

**Table 1:** Sperm quality parameters from *A*. *baerii* (n = 11) and *A*. *schrenckii* (n = 7).

Samples	A Grade Motility (%)	VCL (μm/s)	Fertilization Rate (%)
*A*. *baerii* (AB)			
AB1	52.44±5.70^c^	71.56±4.76^c^	65.99±11.24^a^
AB2	55.21±8.35^b,c^	85.55±4.87^c^	21.67±9.94^b^
AB3	71.79±14.52^a,b,c^	96.76±7.10^a,b^	76.33±6.94^a^
AB4	82.98±6.83^a^	130.63±15.09^a^	72.99±7.49^a^
AB5	57.18±13.04^b,c^	80.81±7.83^c^	45.74±10.08^b,c^
AB6	54.53±14.22^b,c^	81.81±13.41^c^	38.71±4.25^b^
AB7	68.30±23.15^a,b,c^	110.45±25.01^a,b^	81.68±7.10^a^
AB8	57.34±15.97^b,c^	74.02±3.99^c^	28.23±10.49^b^
AB9	43.95±18.86^c^	72.50±25.78^c^	27.16±7.45^b^
AB10	74.02±3.31^a^	142.73±11.83^a^	76.10±9.58^a^
AB11	50.58±26.28^c^	106.19±58.98^a,b^	40.52±14.09^b,c^
*A*. *schrenckii* (ASK)			
ASK1	80.73±1.53^a^	104.62±5.40^a,b^	77.38±7.56^a^
ASK2	77.87±8.48^a^	146.20±28.60^a^	82.36±5.95^a^
ASK3	57.70±17.35^b^	67.48±20.03^b^	22.93±5.80^c^
ASK4	52.11±13.14^b^	69.44±5.80^b^	23.27±9.61^c^
ASK5	69.14±28.33^a,b^	96.57±36.25^a,b^	42.34±5.03^b^
ASK6	71.31±3.53^a,b^	105.69±11.83^a,b^	74.94±5.44^a^
ASK7	51.60±15.94^b^	80.20±20.78^b^	34.33±5.05^b,c^

The means±SEM are given in each column.

The means with different alphabetical superscripts are significantly different (ANOVA-DUNCAN test at *p <* 0.05).

Seven *A*. *schrenckii* males were collected and analysed for sperm motility and fertilization rate (Table 1). The A grade percentage motility of males 1, 2, 5 and 6 was high (69.14–80.73%) and males 3, 4 and 7 was low (34.52–57.70%). The VCL of males 1, 2, 5 and 6 was high (96.57–146.20 μm/s) and males 3, 4 and 7 was low (67.48–80.20 μm/s). The fertilization rate of males 1, 2 and 6 was high (74.94–82.36%), male 5 was medium (42.34%), and males 3, 4 and 7 was low (22.93–34.33%). Combining all parameters under consideration, males 1, 2 and 6 were placed in the high quality group, and males 3, 4 and 7 were placed in the low quality group.

### The proteome of sperm in *A*. *baerii* and *A*. *schrenckii* by iTRAQ analysis

**Fig 1:**
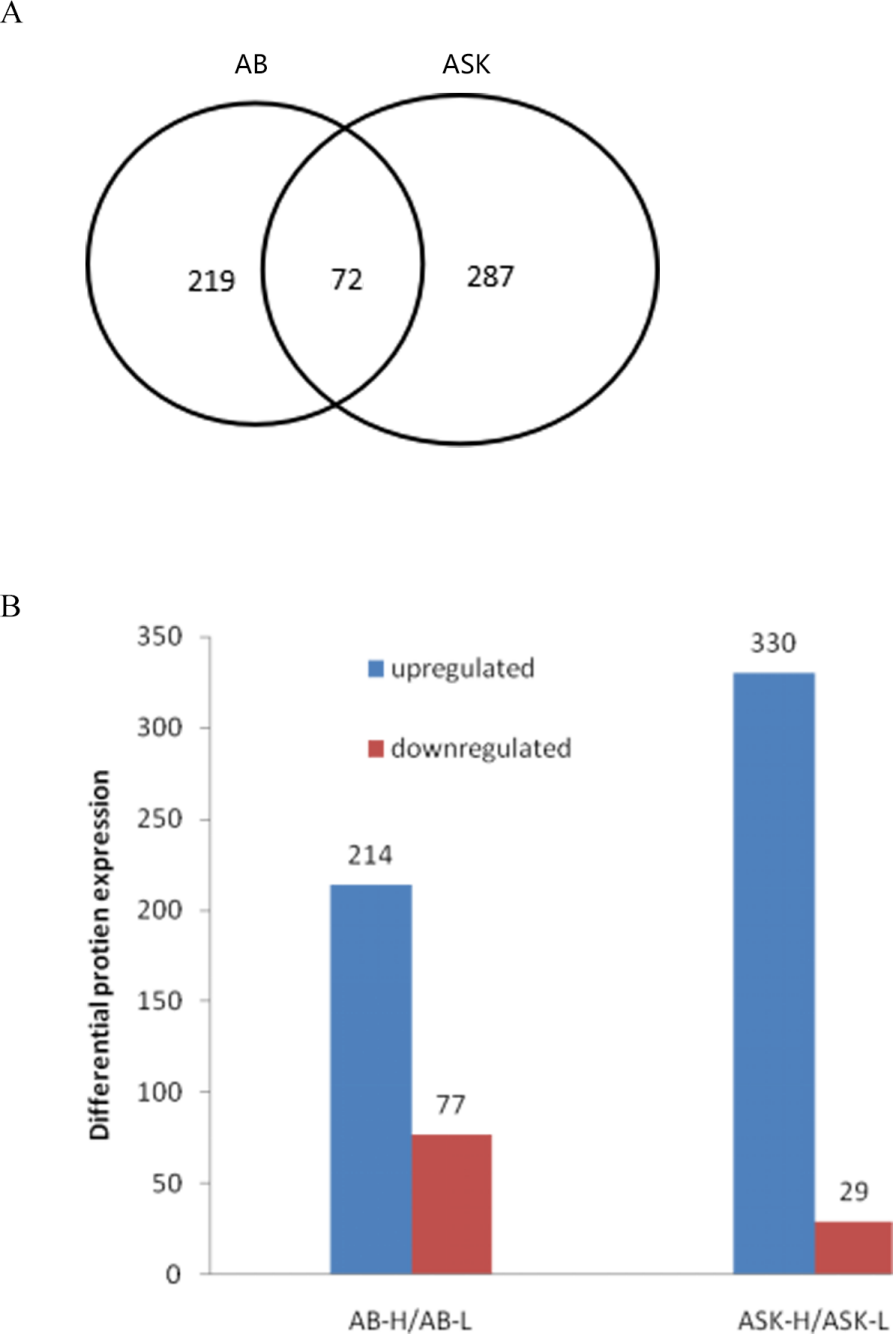
Proteins differentially detected in spermatozoa protein extracts from high quality (H) samples of *A*. *baerii* (AB) and *A*. *schrenckii* (ASK) compared with low quality groups (L). (A) Venn diagram showing the number of different proteins in AB and ASK groups. (B) Number of proteins with significant differences in AB-H/AB-L and ASK-H/ASK-L groups.

In *A*. *baerii*, a total of 1431 proteins were identified from 11856 spectra, of which 9023 were unique, and the proteins accounted for an average of 3.76 peptides, 3.15 unique peptides and 86.92 total spectra. In *A*. *schrenckii*, a total of 1117 proteins were identified from 9123 spectra, of which 8452 were unique, and the proteins accounted for an average of 3.10 peptides, 2.66 unique peptides and 183.87 total spectra. Among the proteins identified, 291 and 359 were differentially expressed in *A*. *baerii* and *A*. *schrenckii*, respectively, and 72 were common to both species (Fig 1A). The quantity levels of 214 proteins were upregulated in samples from the high quality spermatozoa of *A*. *baerii* and 77 were downregulated (Fig 1B). Moreover, 330 proteins were upregulated in the high quality spermatozoa of *A*. *schrenckii* and 29 were downregulated (Fig 1B). Table 2 lists the potential biomarkers related to sperm quality in *A*. *baerii* and *A*. *schrenckii*, along with a description of their functions in the sperm of other animals. Some uncharacterized proteins were not listed here.

**Table 2:** Potential biomarkers related to sperm quality in *A*. *baerii* (AB) and *A*. *schrenckii* (ASK).

GeneID	Nr-annotation	Function	FC in AB	FC in ASK
**cds.Reproduction_Unigene_BMK.**				
73953|m.131636	Prostaglandin E synthase 3 (Cytosolic)	oviductal contractions and sperm transport	2.35	6.85
67825|m.107200	14-3-3 protein beta/alpha-A	binding partners in the seminiferous epithelium, involve in spermatogenesis	1.70	6.57
57410|m.74855	Ckmt1 protein	sperm motility	1.57	4.25
58127|m.76641	Argininosuccinate synthase	arginine metabolism	1.66	3.82
69611|m.113862	Phosphoglycerate kinase	spermatogenesis, and required for normal sperm motility and fertility	1.63	3.76
81168|m.172453	L-lactate dehydrogenase B-A chain	glucose metabolism, support energy for motility and fertilization	2.69	3.71
57711|m.75610	Superoxide dismutase [Cu-Zn]	antioxidant	1.59	3.68
62099|m.87878	Heat shock 10 protein 1 (Chaperonin 10)	protein folding and the assemblage of multimeric protein complexes, involved in sperm-zona pellucida interaction	1.85	3.66
63509|m.92502	Ubiquitin carboxyl-terminal hydrolase	protein folding, sperm- zona pellucida interactions and antipolyspermy defence	1.63	3.54
66959|m.104072	Proteasome (Prosome, macropain) 26S subunit, non-ATPase, 13	protein folding, sperm penetration	1.62	3.54
67897|m.107468	Protein disulphide-isomerase (Fragment)	maturation, fertility	1.99	3.46
58850|m.78570	Nuclear transport factor 2	responsible for nuclear import of Ran	2.06	3.45
52904|m.64459	ER membrane protein complex subunit 10	—	1.66	3.05
70460|m.117146	Glycerol-3-phosphate dehydrogenase [NAD(+)]	carbohydrate metabolism and lipid metabolism, sperm capacitation,	1.52	2.91
64273|m.95032	Translationally controlled tumour protein homologue	spermatogenesis, apoptosis, cellular differentiation, and in the control of sperm functions	1.63	2.84
51647|m.61888	Fascin	actin filament-binding protein, elongation of the spermatid head and in microfilament rearrangements during spermatogenesis	2.15	2.74
58359|m.77237	Carbonyl reductase 1	oxidoreductase, sperm-zona pellucida interaction and fertilization	2.94	2.74
67622|m.106450	Isocitrate dehydrogenase [NADP]	TCA cycle, sperm capacitation	1.82	2.68
58128|m.76647	Aspartate aminotransferase	amino acid metabolism	2.89	2.68
78920|m.156098	Exportin-2	nucleocytoplasmic transport during spermatogenesis	2.35	2.68
78521|m.153973	Heat shock 60 kD protein 1 (Chaperonin)	protein folding, immune response	2.75	2.55
60770|m.83998	Proteasome subunit beta type	protein folding	1.50	2.55
66148|m.101273	Succinyl-CoA:3-ketoacid-coenzyme A transferase	energy metabolism	3.25	2.42
71761|m.122354	T-complex protein 1 subunit delta	binding of sperm to zona pellucida	1.67	2.40
71098|m.119623	3-hydroxyisobutyryl-CoA hydrolase, mitochondrial	valine metabolism	1.94	2.26
34078|m.35467	Ubiquitin A-52 residue ribosomal protein fusion product 1	—	1.92	2.20
80484|m.166275	Protein DDI1 homologue 2	—	1.57	2.15
68576|m.109959	Glucose-6-phosphate 1-dehydrogenase	energy metabolism	1.84	2.13
53340|m.65415	ATPase inhibitor B, mitochondrial	respiration	2.49	2.08
68031|m.107921	Si:dkey-46a12.1	amino acid metabolism, regulation of nitric oxide biosynthetic process	3.04	1.93
59440|m.80152	Brain-subtype creatine kinase	energy homeostasis	1.53	1.91
60160|m.82109	Malate dehydrogenase	energy metabolism	1.98	1.88
77538|m.148765	Pyruvate dehydrogenase E1 component subunit alpha	energy metabolism	1.61	1.78
75572|m.139013	Carnitine O-palmitoyltransferase 2, mitochondrial	regulation of mitochondrial fatty acid oxidation	1.60	1.77
61077|m.84834	Solute carrier family 25 (Mitochondrial carrier adenine nucleotide translocator), member 6	energy transport, sperm maturation	1.58	1.72
74964|m.136216	Neuraminidase 1	glycosphingolipid metabolism, Sialic acid metabolism, spermatogenesis	1.71	1.69
62864|m.90398	Branched-chain-amino-acid aminotransferase	amino acid metabolism	1.94	1.67
54493|m.68016	Fumarate hydratase, mitochondrial	TCA cycle, sperm capacitation	1.72	1.65
79580|m.160034	Dihydrolipoyl dehydrogenase	energy metabolism, involve in acrosome reaction, capacitation, fertilization and motility	1.87	1.61
68628|m.110178	Succinate dehydrogenase [ubiquinone] flavoprotein subunit, mitochondrial	TCA cycle, sperm motility	2.58	1.57

Note: “—” means the function is unknown in sperm. FC means fold change.

### Functional classification and interaction of differentially expressed proteins

**Fig 2:**
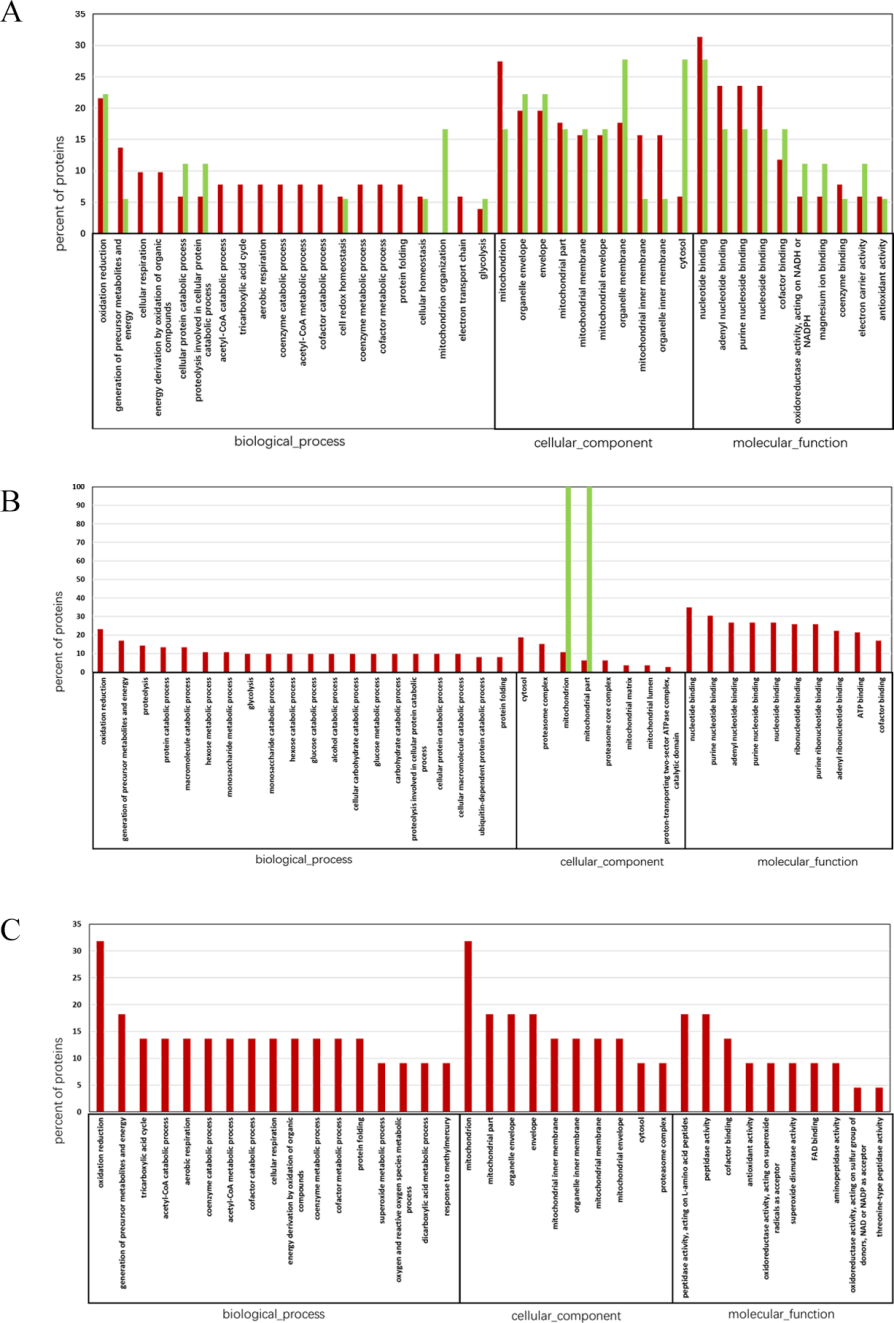
Gene ontology (GO) of the differentially expressed proteins identified in spermatozoa protein extracts from high quality samples compared with low quality groups. (A) *A*. *baerii*. (B) *A*. *schrenckii*. (C) overlapped results from both species. Red columns represent upregulated proteins and green column represents downregulated proteins.

To understand the functional basis of the differentially expressed proteins identified by iTRAQ technology, we used gene ontology (GO) to analyse and classify the molecular functions of the significantly altered proteins. Proteins were sorted into categories according to their ontology as determined from their GO annotation terms (Fig 2). In *A*. *baerii* (Fig 2A), 113 annotated genes were assigned to 40 subcategories. Of these, the two most prominent biological processes were oxidation reduction (representing 21.57% of the upregulated proteins and 22.22% of the downregulated proteins), the generation of precursor metabolites and energy (representing 13.73% of the upregulated proteins and 5.55% of the downregulated proteins). Most proteins were localized to the mitochondria (27.45% of upregulated proteins and 16.67% of downregulated proteins), the organelle envelope and the envelope (19.61% of upregulated proteins and 22.22% of downregulated proteins in both parts). Approximately 31.37% of upregulated proteins and 27.78% of downregulated proteins were associated with nucleotide binding, followed by adenyl nucleotide binding, purine nucleoside binding, and nucleoside binding (23.53% of upregulated proteins and 16.67% of the downregulated proteins in these three categories). In *A*. *schrenckii* (Fig 2B), 113 annotated genes were assigned to 38 subcategories. Of these, the two most prominent biological processes were oxidation reduction (representing 23.21% of upregulated proteins), generation of precursor metabolites and energy (representing 16.96% of upregulated proteins). Most proteins were localized to the mitochondria (10.71% of upregulated proteins and 100% of downregulated proteins) and mitochondria part (6.25% of upregulated proteins and 100% of downregulated proteins). Approximately 34.82% of upregulated proteins were associated with nucleotide binding, followed by purine nucleoside binding (30.36% of upregulated proteins). To compare the common functional annotation, the differentially expressed proteins in both species were aligned before the GO analysis (Fig 2C). The common differentially expressed proteins for both species were all upregulated. Of these, oxidation reduction (31.82%) and generation of precursor metabolites and energy (18.18%) were the most common biological processes. The mitochondria contained 31.82% of the proteins, 13.64% of the proteins were localized in the parts of the mitochondria, the organelle envelope and the envelope. Peptidase activity characterized 18.18% of the proteins followed by cofactor binding (13.64%).

**Fig 3:**
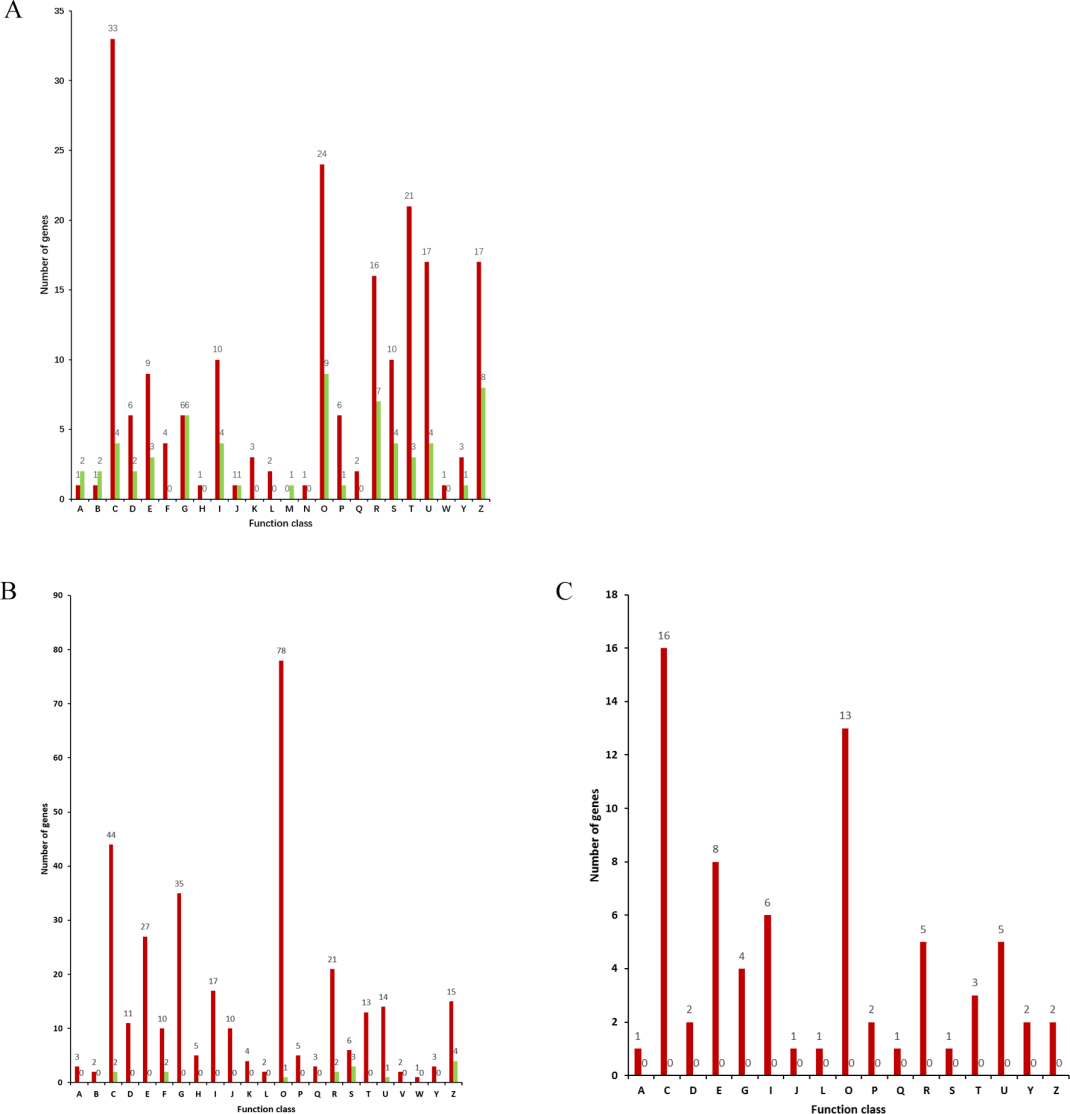
Cluster of orthologous group (COG) classification of the differentially expressed proteins identified in spermatozoa protein extracts from high quality samples compared with low quality groups. (A) *A*. *baerii*. (B) *A*. *schrenckii*. (C) overlapped results from both species. Red columns represent upregulated proteins and green columns represent downregulated proteins. Classification: Information storage and processing [J] Translation, ribosomal structure and biogenesis [A] RNA processing and modification [K] Transcription [L] Replication, recombination and repair [B] Chromatin structure and dynamics Cellular processes and signalling [D] Cell cycle control, cell division, chromosome partitioning [Y] Nuclear structure [V] Defence mechanisms [T] Signal transduction mechanisms [M] Cell wall/membrane/envelope biogenesis [N] Cell motility [Z] Cytoskeleton [W] Extracellular structures [U] Intracellular trafficking, secretion, and vesicular transport [O] Posttranslational modification, protein turnover, chaperones Metabolism [C] Energy production and conversion [G] Carbohydrate transport and metabolism [E] Amino acid transport and metabolism [F] Nucleotide transport and metabolism [H] Coenzyme transport and metabolism [I] Lipid transport and metabolism [P] Inorganic ion transport and metabolism [Q] Secondary metabolites biosynthesis, transport and catabolism Poorly characterized [R] General function prediction only [S] Function unknown.

All of the differentially expressed proteins were submitted to the Cluster of Orthologous Groups of Proteins (COG) database to search for functional predictions and classification, and 257, 346 and 73 proteins from the high quality spermatozoa of *A*. *baerii*, *A*. *schrenckii* and the overlapped data from both species, respectively, were assigned to 25 COG functional categories (Fig 3). In *A*. *baerii*, the upregulated proteins were predominantly grouped in the categories “RNA processing and modification”; “Posttranslational modification, protein turnover, chaperones”; “Signal transduction mechanisms”; “Intracellular trafficking, secretion, and vesicular transport”; and “Cytoskeleton”; while the downregulated proteins were predominantly grouped in the categories “Posttranslational modification, protein turnover, chaperones” and “Cytoskeleton”. In *A*. *schrenckii*, the upregulated proteins were predominantly grouped in the categories “Posttranslational modification, protein turnover, chaperones”; “Energy production and conversion”; “Carbohydrate transport and metabolism” and “Amino acid transport and metabolism”; while the downregulated proteins were predominantly grouped in the category “Cytoskeleton”. The overlapped proteins from both species were all upregulated and predominantly clustered in the categories “Energy production and conversion”; “Posttranslational modification, protein turnover, chaperones” and “Amino acid transport and metabolism.”

**Fig 4:**
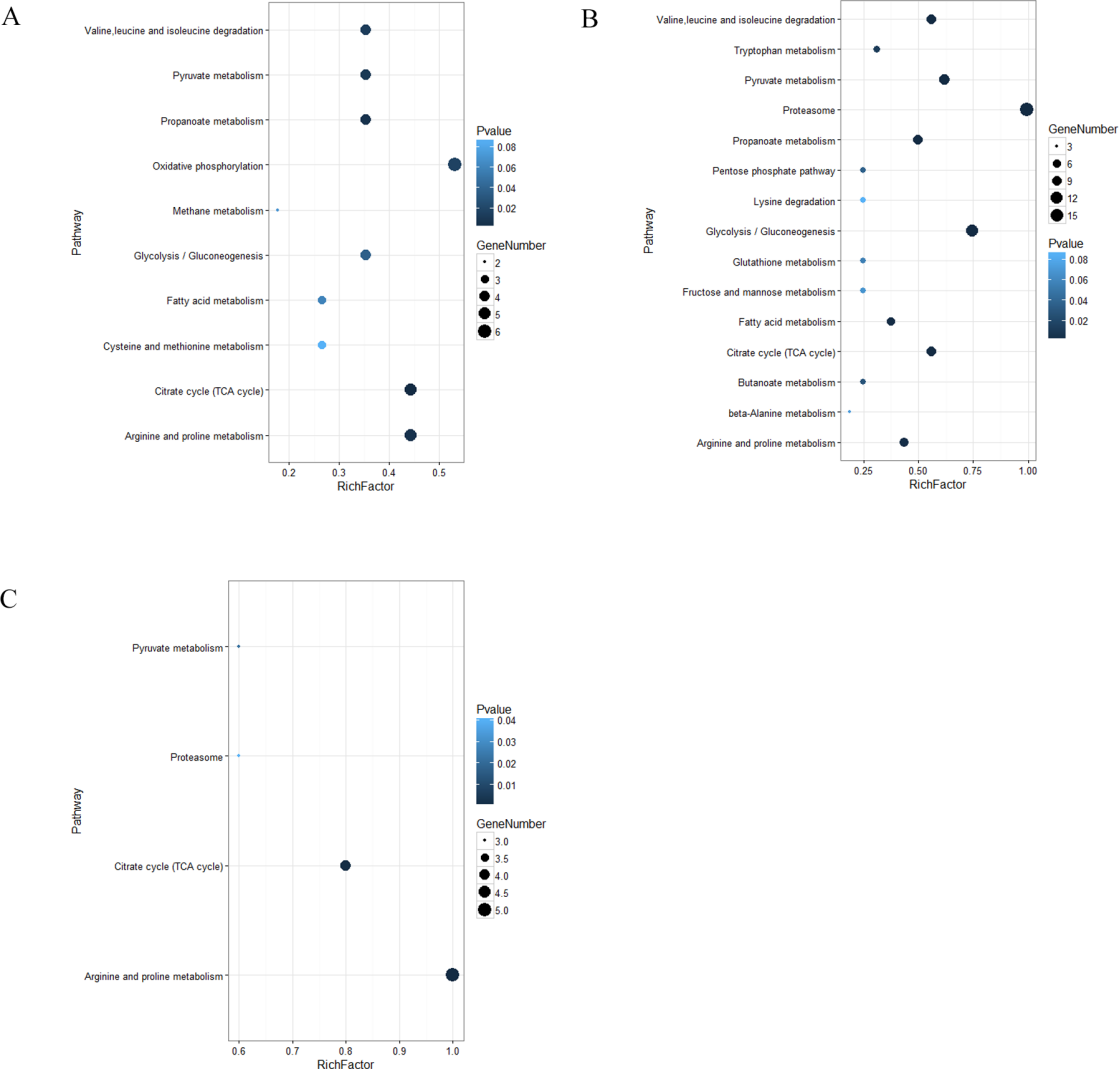
KEGG pathway enrichment of the differentially expressed proteins identified in spermatozoa protein extracts from high quality samples compared with low quality groups. (A) *A*. *baerii*. (B) *A*. *schrenckii*. (C) overlapped results from both species.

**Fig 5:**
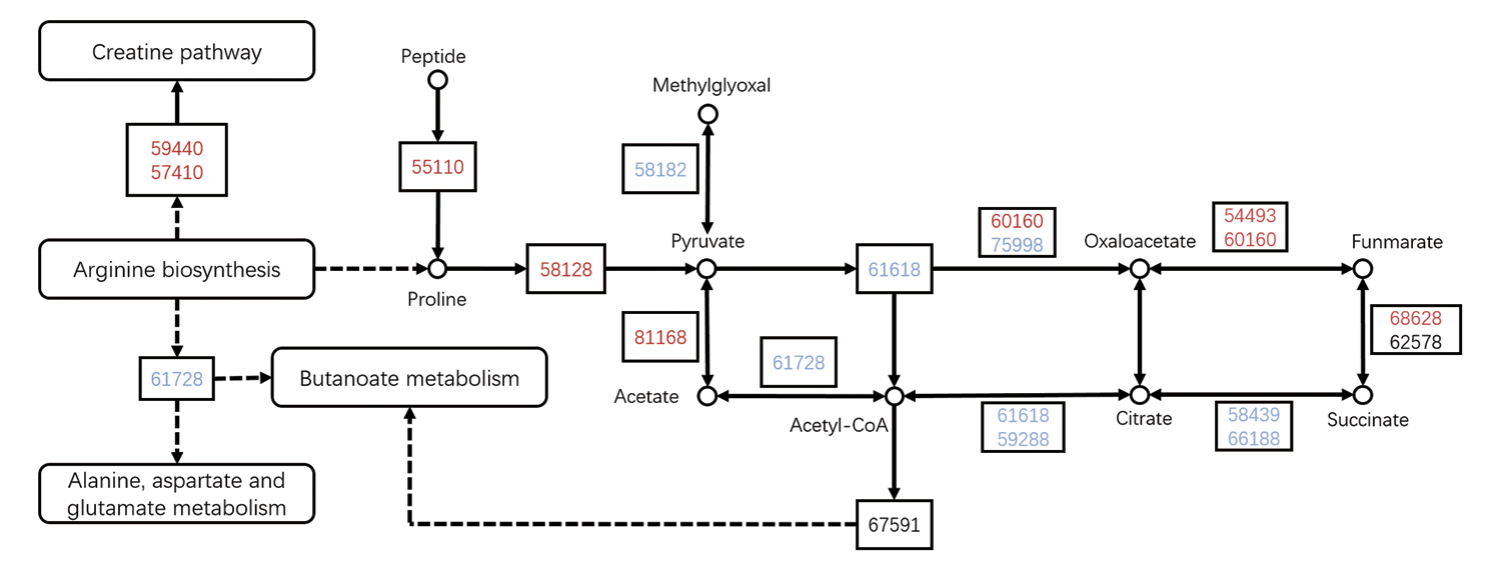
Pathway analysis using the Kyoto Encyclopedia of Genes and Genomes (KEGG) Pathway database. Proteins from the pathways of Arginine and proline metabolism, the Citrate cycle (TCA cycle) and Pyruvate metabolism are shown. The red number represents proteins common to *A*. *baerii* and *A*. *schrenckii*. The blue number represents proteins unique to *A*. *schrenckii*. The black number represents proteins unique to *A*. *baerii*.The protein names are listed in Table 3.

A pathway enrichment analysis indicates the significantly enriched pathways for the differentially expressed proteins compared to all proteins identified, and it uses the KEGG pathway as a unit and the hypergeometric test. A significant enrichment analysis can determine the main biochemical pathways and the signal transduction pathways that involve the differentially expressed proteins. In *A*. *baerii* (Fig 4A), the pathways containing the most differentially expressed proteins were “Citrate cycle (TCA cycle)”, followed by “Arginine and proline metabolism”, “Propanoate metabolism”, “Pyruvate metabolism”, “Valine, leucine and isoleucine degradation”, “Oxidative phosphorylation” and “Glycolysis / Gluconeogenesis”. In *A*. *schrenckii* (Fig 4B), the pathways containing the most differentially expressed proteins were “Proteasome”, followed by “Glycolysis / Gluconeogenesis”, “Pyruvate metabolism”, “Citrate cycle (TCA cycle)”, “Valine, leucine and isoleucine degradation”, “Propanoate metabolism”, and “Fatty acid metabolism”. The overlapped proteins from both species were involved in the pathways “Arginine and proline metabolism”, “Citrate cycle (TCA cycle)”, “Pyruvate metabolism”, and “Proteasome” (Fig 4C). Fig 5 and Table 3 shows the common and unique proteins for both species involved in the three pathways “Arginine and proline metabolism”, “Citrate cycle (TCA cycle)” and “Pyruvate metabolism”.

**Table 3:** Protein list.

GeneID	Nr-annotation
cds.Reproduction_Unigene_BMK.	
59440|m.80152	Brain-subtype creatine kinase
57410|m.74855	Ckmt1 protein
55110|m.69344	Uncharacterized protein
58128|m.76647	Aspartate aminotransferase
81168|m.172453	L-lactate dehydrogenase B-A chain
60160|m.82109	Malate dehydrogenase
54493|m.68016	Fumarate hydratase, mitochondrial
68628|m.110178	Succinate dehydrogenase [ubiquinone] flavoprotein subunit, mitochondrial
58182|m.76758	Lactoylglutathione lyase
61728|m.86812	Aldehyde dehydrogenase family 9 member A1-A
61618|m.86458	Pyruvate dehydrogenase (Lipoamide) beta
75998|m.140962	Malic enzyme
59288|m.79761	Citrate synthase, mitochondrial
58439|m.77455	Isocitrate dehydrogenase [NAD] subunit, mitochondrial
66188|m.101375	Succinyl-CoA ligase subunit beta
67591|m.106318	Acetyl-CoA acetyltransferase, mitochondrial
62578|m.89440	Succinate dehydrogenase [ubiquinone] iron-sulphur subunit, mitochondrial

### Validation of the quantitative proteomic analysis by western blotting

**Fig 6:**
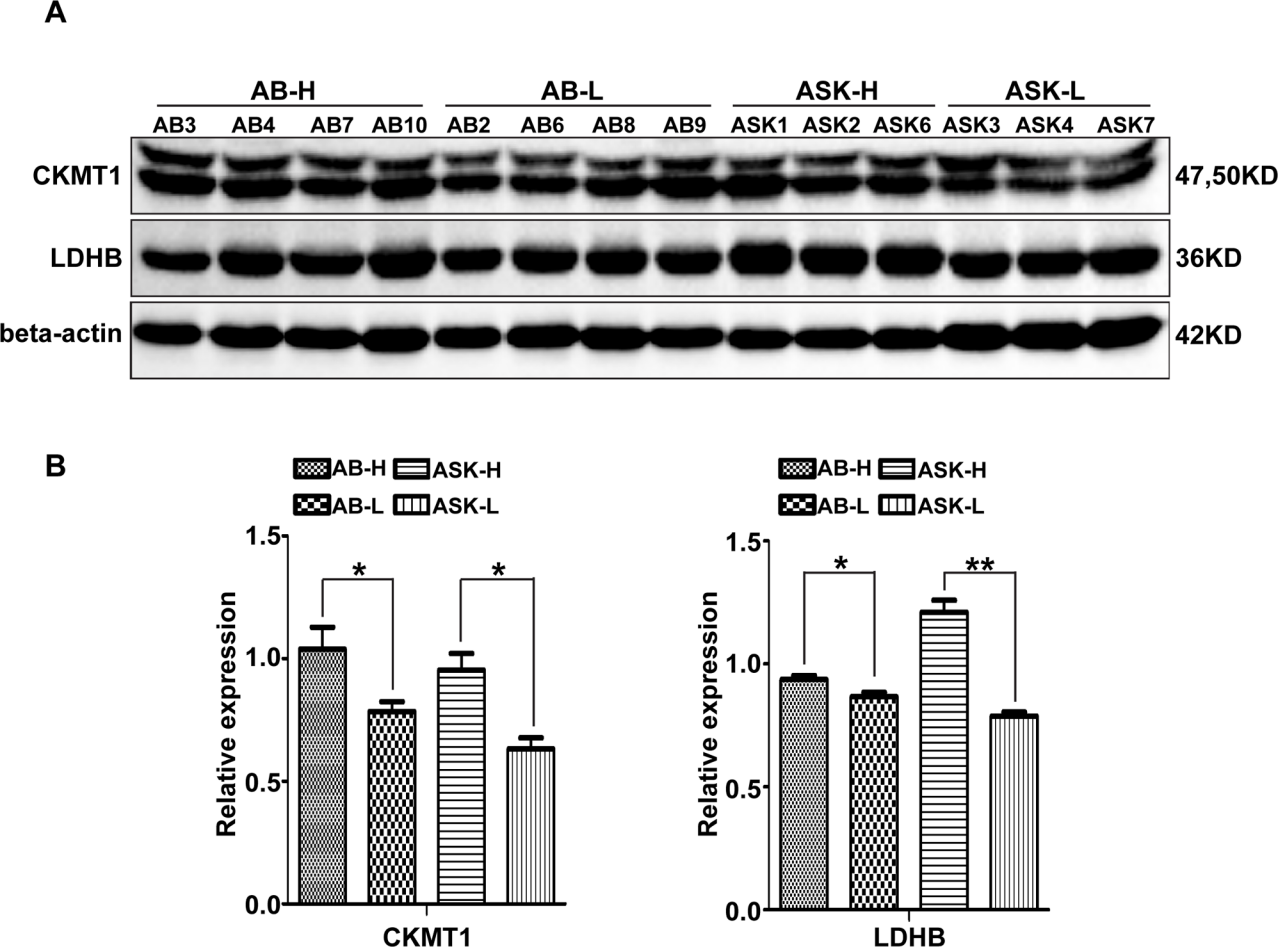
Western blot validation of CKMT1 and LDHB proteins. (A) CKMT1 and LDHB proteins in high quality (AB3, AB4, AB7, and AB10) and low quality (AB2, AB6, AB8, and AB9) samples of *A*. *baerii* spermatozoa, as well as high quality (ASK1, ASK2, and ASK6) and low quality (ASK3, ASK4, and ASK7) samples of *A*. *schrenckii* spermatozoa. Beta-actin was used as the loading control. (B) Quantification of protein levels in high and low quality of spermatozoa from *A*. *baerii* and *A*. *schrenckii* based on western blot analysis. *p<0.05, **p<0.01.

Western blot analysis was carried out to validate the levels of differentially expressed proteins (Fig 6A and 6B). We selected the proteins CKMT1 and LDHB from the pathways of Arginine and proline metabolism, TCA cycle and Pyruvate metabolism. The changes in protein levels as determined by the western blot analysis were consistent with the variations indicated by the iTRAQ analysis. CKMT1 and LDHB were significantly higher expressed in high quality than in low quality spermatozoa from *A*. *baerii* and *A*. *schrenckii*, respectively (p<0.05).

## Discussion

In the current study, we compared the spermatozoa proteome from a group of sturgeons with high quality sperm to sturgeons with low quality sperm to help to improve the prediction of sperm fertility after spermiation, significantly contribute to an improvement in sturgeon reproductive techniques, and aid in the selection of high-quality fertile sperm for preservation.

We divided the spermatozoa into high and low quality groups according to sperm motility and the fertilization rate. We identified 1431 and 1117 proteins in *A*. *baerii* and *A*. *schrenckii*, respectively, where 291 and 359 of which had a >1.5-fold difference in quantity levels in the high quality group compared to the low quality group. Based on the spectral counts, we identified 214 and 330 overexpressed proteins and 77 and 29 underexpressed proteins, respectively, in the low quality *A*. *baerii* and *A*. *schrenckii* groups compared to the high quality groups. Of these, 72 proteins were common in both species, and all were overexpressed in the high quality groups. The cellular distribution of the differentially expressed proteins identified in the low quality spermatozoa as ascertained by the GO analysis suggested that the majority of the proteins were localized to the mitochondria and parts of the mitochondria in both species. An examination of the association of the differentially expressed proteins with biological processes indicated that the majority of the proteins were involved in generation of precursor metabolites and energy as well as oxidation reduction, and their molecular functions were mostly associated with peptidase activity and cofactor binding.

Several energy metabolism and respiratory chain enzymes were more abundant in the most fertile males. This finding is in accordance with the high energy level needed by sperm for their long and active journey in the male, followed by fertilization of the egg [15]. The tricarboxylic acid cycle (TCA) is a key metabolic pathway for energy supply that unifies carbohydrate, fat, and protein metabolism. The electron transport chain in the mitochondria is the site of oxidative phosphorylation where the NADH and succinate generated in the TCA cycle are oxidized to produce ATP [16]. ATP is required for sperm motility, hyperactivation, capacitation, acrosome reaction, and subsequent fertilization [17–18]. High concentrations of glucose, pyruvate, and lactate are present in the mammalian oviductal fluid [19] and are commonly utilized by spermatozoa as energy substrates [20–22]. The metabolism of these substrates can either go through the lactate dehydrogenase reduction of pyruvate to lactate, which regenerates NAD^+^ for glycolysis, or the reverse reaction, which generates the pyruvate that is incorporated into the TCA cycle for OXPHOS [23]. Pyruvate and lactate are transported by monocarboxylate transporters (MCTs) into the spermatozoa and metabolized via electron transfer in the respiratory chain to support sperm motility in goats, stallions and fish species [24–26]. The mechanism of ATP production for various metabolic substrates has been shown to vary between species [27]. Free amino acids have also been detected in spermatozoa and the seminal plasma of mammals and fish species [28–29]. Amino acids were catabolized by transamination, decarboxylation and oxidative deamination. The resulting amino acid fragments were theoretically used as a fuel to provide energy, mostly via the TCA cycle or to serve as a basis for various biosynthetic processes [30]. The amino acid composition and metabolism vary among different species. Arginine and proline metabolism is a central pathway for the biosynthesis of the amino acids arginine and proline from glutamate, which has also been identified in the present study. In our study, we found that high quality sturgeon sperm over expressed proteins involved in Arginine and proline metabolism (Brain-subtype creatine kinase, CKMT1 protein, and Aspartate aminotransferase), Pyruvate metabolism (L-lactate dehydrogenase B-A chain and Malate dehydrogenase), and the TCA cycle (Malate dehydrogenase, Fumarate hydratase, and Succinate dehydrogenase [ubiquinone] flavoprotein subunit), which suggests that Arginine and proline metabolism and Pyruvate metabolism are important metabolic pathways for supporting energy in sturgeon spermatozoa via the TCA cycle (Fig 5, Table 3). Metabolic enzymes expressed at a lower level in low quality spermatozoa may affect sperm motility and subsequently sperm fertility.

Energy substrates have been used in sperm medium for storage and cryopreservation [25–26, 28]. However, sperm medium should be formulated with specific energy requirements in mind, as it appears that there must be a delicate balance of mitochondrial inputs in order to achieve optimal mitochondrial functionality. Exogenous lactate and pyruvate play a vital role in stallion spermatozoa mitochondrial functionality and motility in a dose-dependent manner, as spermatozoa in these media operate at a very high level of bioenergetic capability due to their high rate of energy metabolism [26]. Supplementation with arginine stimulates metabolic the activity of spermatozoa in goats [31] and motility in rabbits [32] and human spermatozoa in vitro [33–34]. Arginine also serves as a source of NO, inducing capacitation and an acrosome reaction in bull spermatozoa [35]. Proline has been reported to improve motility and velocity and to preserve the structural and functional integrity of biological membranes during freezing and thawing [36–38] probably by protecting them from free-radical-induced damage [39]. Proline acts as an antioxidant and reduces lipid peroxidation, and it can penetrate the spermatozoa and inhibit intracellular ice formation. In the future, we could test the effects of lactate, pyruvate, arginine and proline supplied in the medium for the storage of sturgeon sperm.

Other metabolic enzymes such as glycerol-3-phosphate dehydrogenase, phosphoglycerate kinase, succinyl-CoA:3-ketoacid-coenzyme A transferase, 3-hydroxyisobutyryl-CoA hydrolase, si:dkey-46a12.1, solute carrier family 25, neuraminidase 1, branched-chain-amino-acid aminotransferase and dihydrolipoyl dehydrogenase, which are involved in carbohydrate, lipid and amino acid metabolism, were also found to be over expressed in high quality sturgeon spermatozoa. The involvement of different enzymes associated with many energy production pathways and ATP production in spermatozoa probably reflect the excitation of sperm cells to deliver the maximum energy required for high motility and fertility.

The higher energy activity is also accompanied by greater activity of the oxido-reduction enzymes and protection from reactive oxygen species, which have been associated with a loss of sperm motility, a decreased capacity for sperm-oocyte fusion and a loss of fertility [16]. For example, superoxide dismutase [Cu-Zn] has been shown to be an important antioxidant defence in fish [40] and mammalian [41] sperm. Carbonyl reductase 1 is a cytosolic, monomeric, NADPH-dependent oxidoreductase with broad specificity for carbonyl compounds and a general tissue distribution [42]. Carnitine palmitoyltransferases are key components in the mitochondrial transport of long-chain fatty acids and provide an important mechanism for the regulation of mitochondrial fatty acid oxidation in all body tissues [43]. Many antioxidative and redox enzyme genes are expressed and aggressively protect gametes and embryos in the reproductive system [44]. Thus, our results strongly suggest that the enzymes, superoxide dismutase [Cu-Zn], carbonyl reductase 1 and carnitine palmitoyltransferases, play an important role in scavenging or detoxifying excess reactive oxygen species (ROS) in sturgeon sperm (Table 2).

Ubiquitin-proteasome-dependent proteolysis has been implicated in the control of mammalian gametogenesis and fertilization [45–48]. It plays an important role in selectively degrading and recycling proteins in many basic cellular processes, including but not limited to differentiation, cell cycle control, apoptosis, and the immune response [49]. The 26S proteasome, which is a multi-subunit protease specific for postranslationally modified substrate proteins by ubiquitination, has been implicated in acrosomal function and sperm-zona pellucida (ZP) penetration during mammalian fertilization. Ubiquitin C-terminal hydrolases (UCHs) are responsible for the removal of polyubiquitin chains during substrate priming for proteasomal proteolysis [50]. A ubiquitin system has also been found in carp (*Cyprinus carpio* L) sperm and was involved in sperm motility [51]. In our study, it is strongly suggested that UCHs and proteasome play an important role in the regulation of spermatogenesis and sperm quality control, demonstrating the importance of ubiquitin-proteasome-dependent proteolysis in sperm maturation and fertilization in sturgeon since both were expressed at higher levels in high quality spermatozoa (Table 2).

Heat shock proteins (HSPs) are essential stress proteins that foster cell survival under adverse environmental conditions. At the cellular level, HSPs function as chaperones and important regulators of differentiation, cell division and apoptosis [52]. Recently, HSPs (including HSP 10 and HSP 60) have been shown to be present on the surface of spermatozoa and may mediate the assembly of a protein receptor complex for the recognition of the zona pellucida [53–54]. Thus, the presence of HSP is believed to be an essential factor for sperm fertility. Further studies should focus on the characterization of HSPs, in particular, their localization in sturgeon spermatozoa and their function in fertilization.

According to the previous study, 14-3-3 and its binding partners are regulators of protein–protein interactions during spermatogenesis [55]. The chaperonin-containing T-complex protein 1 is a member of the class II chaperonins localized in the centrosomes and microtubules during spermatogenesis and discarded in the residual bodies at spermiation and was involved in sperm-egg fusion in mammals [56]. Fascin, an actin filament-binding protein, may function in the terminal elongation of the spermatid head and in microfilament rearrangements during spermatogenesis [57]. In summary, our results suggest that several proteins play a vital role in sperm maturation during spermatogenesis and affect sturgeon sperm quality. Further studies are necessary to clarify the roles of those proteins.

The comparative proteomic analysis carried out on low and high quality sturgeon spermatozoa in this study showed that sperm quality (i.e., motility and fertility) is associated with the expression levels of certain proteins. Proteins upregulated in high quality spermatozoa are mainly involved in metabolic pathways for the generation of precursor metabolites and energy. Others are associated with oxidation reduction, ubiquitin-proteasome-dependent proteolysis, chaperones and binding activity. We believe that our findings contribute appreciably to increasing basic information on the proteins involved in sturgeon sperm biology. Moreover, such information will shed more light on the potential role of these proteins in sturgeon spermatozoa and may help to develop appropriate reproductive technologies for the genetic conservation and management of this threatened species.
